# Supplementary material for: Genetic ancestry plays a central role in population pharmacogenomics
Source: Commun Biol. 2021 Feb 5;4:171. doi: 10.1038/s42003-021-01681-6 (PMC7864978; doi:10.1038/s42003-021-01681-6)
Supplement: Supplementary file 2 — Supplementary Information [file 42003_2021_1681_MOESM2_ESM.pdf]

Supplementary Information in "Genetic ancestry plays a central role in population pharmacogenomics"

Hsin-Chou Yang[1,2,3,*], Chia-Wei Chen[1], Yu-Ting Lin[1], and Shih-Kai Chu[1]

[1]Institute of Statistical Science, Academia Sinica, Taipei 11529, Taiwan

[2]Institute of Statistics, National Cheng Kung University, Tainan 70101, Taiwan

[3]Institute of Public Health, National Yang-Ming University, Taipei 11221, Taiwan

*Corresponding author: Hsin-Chou Yang, Institute of Statistical Science, Academia Sinica. No. 128, Sec. 2, Academia Road, Nankang 11529, Taipei, Taiwan

(Fax) 886-2-27886833

(Tel) 886-2-27875686

(E-mail) hsinchou@stat.sinica.edu.tw

Table Contents

**Supplementary Note 1.**	Ultrahigh-dimensional principal component analysis …….…...	**3**
**Supplementary Note 2.**	Genetic ancestry pharmacogenomic database …………………	**4**
**Supplementary Note 3.**	Top AIMs with the maximum difference in allele frequencies across the studied continental ancestry groups and within a specific continental ancestry group……………………………..	**5**
**Supplementary Figure 1.**	Principal component analysis of whole-genome homozygosity intensity of 2,504 individuals ………………..............................	**6**
**Supplementary Figure 2.**	Parallel coordinates plot of 10-fold cross validations …………	**7**
**Supplementary Figure 3.**	Overlay line graph of 10-fold cross validations for training and testing accuracy ……………………………………………...	**8**
**Supplementary Figure 4.**	Staked-bar/Box-whisker plot of the best prediction panel ….....	**9**
**Supplementary Figure 5.**	Marker impact plot of the best prediction panel ………..........	**10**
**Supplementary Figure 6.**	Multidimensional scaling plot of 2,504 individuals and 32 predictors ………………………………………………….....	**11**
**Supplementary Figure 7.**	Ideograms of genome-wide homozygosity intensity	**12**
**Supplementary Figure 8.**	Global genotype distribution of rs1801133 on *MTHFR* ….........	**13**

Supplementary Note 1. — Ultrahigh-dimensional principal component analysis

Let $\boldsymbol{A}$ denote a data matrix with m rows and n columns. Here, m indicates the number of SNVs and n indicates the number of individuals. The element at the ith row and jth column indicates the individual-level allele frequency, coded by 0, 0.5, or 1, of the jth SNV in the ith individual[1,2]. A single value decomposition derives

$$\boldsymbol{A} = \boldsymbol{U\Sigma V}^T, \tag{1}$$

where $\boldsymbol{U}$ is a $m \times m$ orthogonal matrix, $\boldsymbol{V} = [\boldsymbol{v}_1, \boldsymbol{v}_2, \cdots, \boldsymbol{v}_n]$ is a $n \times n$ orthogonal matrix, and $\boldsymbol{\Sigma}$ is a $m \times n$ matrix made by the upper and lower blocks (submatrices). The upper block $\boldsymbol{D} = diag\{\lambda_i, i = 1, \cdots, n\}$ is a $n \times n$ diagonal matrix and the lower block is a $(m - n) \times n$ zero matrix. Pair $\{(\boldsymbol{v}_i, \lambda_i), i = 1,, \cdots, n\}$ indicate the eigenvectors and eigenvalues of the data matrix $\boldsymbol{A}$.

Because the dimension m is ultrahigh, it makes the matrix manipulation of $\boldsymbol{U}$ intractable. In most cases, we are only interested in the top singular vectors (e.g., the first q vectors). For a dimension reduction, we can consider a rank-q singular value decomposition. Let

$$\boldsymbol{M} = \boldsymbol{A}^T\boldsymbol{A}. \tag{2}$$

It is easy to derive that the eigenvalues of $\boldsymbol{M}$ are the squares of the singular values of $\boldsymbol{A}$. The eigenvectors of $\boldsymbol{M}$ are the right singular vectors of $\boldsymbol{A}$ and represent the effect of individuals. Similarly, we can also obtain the first q left singular vectors of $\boldsymbol{A}$ by $\boldsymbol{AV}_q\boldsymbol{D}_q^{-1}$, where $\boldsymbol{V}_q = [\boldsymbol{v}_1, \boldsymbol{v}_2, \cdots, \boldsymbol{v}_q]$ and $\boldsymbol{D}_q = diag\{\lambda_i, i = 1, \cdots, q\}$, and represent the effect of single nucleotide variation.

To further reduce the matrix manipulation for $\boldsymbol{AV}_q\boldsymbol{D}_q^{-1}$, a block-wise singular value decomposition is proposed. We partition the matrix $\boldsymbol{A}$ into p submatrices (blocks) as follows:

$$\boldsymbol{A} = \begin{bmatrix} \boldsymbol{A}_1 \\ \boldsymbol{A}_2 \\ \vdots \\ \boldsymbol{A}_p \end{bmatrix} \tag{3}$$

where $\boldsymbol{A}_i$ is a $k_i \times n$ matrix and $\sum_{i=1}^{p} k_i = m$. Finally, $\boldsymbol{M}$ in Equation (2) is replaced with $\boldsymbol{M} = \boldsymbol{A}^T\boldsymbol{A} = \sum_{i=1}^{p} \boldsymbol{A}_i^T \boldsymbol{A}_i$ and the aforementioned procedure for $\boldsymbol{AV}_q\boldsymbol{D}_q^{-1}$ is similarly applied to reduce the matrix manipulation.

Compared to the original algorithm of principal component analysis, the proposed ultrahigh-dimensional principal component analysis provides an efficient alternative in terms of computational memory and the results are the same (without a loss of estimation accuracy). It is especially suitable for the large data set in a whole-genome sequencing data analysis.

Supplementary Note 2. — Genetic ancestry pharmacogenomic database

Genetic Ancestry PhD is divided into three components: Introduction, Ancestry Informative Marker (AIM), and Ancestry Informative Gene (AIG). The Introduction page provides a briefing about this website.

The Ancestry Informative Marker (AIM) page contains three pages: Intro, AF, and Pharmacogenomics (AIM). The Intro page provides a briefing about the data sheet for AIM. The AF page provides allele frequency of AIMs associated with the studied ancestry groups. The results are shown according to the studied ancestry groups (AFR, AMR, EAS, EUR, SAS, and whole-continents) and followed by populations in each ancestry group. For each AIM, the minor allele (in parenthesis) and its frequency in each population are provided; the analysis type (i.e., wg, rare, common, or common&rare) is provided; the adjusted *P* value determined using the false discovery rate is provided. The Pharmacogenomics (AIM) page provides the biological importance and drug information of AIMs of interest. This information includes nucleotide change, gene symbol, functional annotation, gene regulation, biotransformation, ADME class, and drug information.

The Ancestry Informative Gene (AIG) page contains three pages: Intro, HI, and Pharmacogenomics (AIG). The Intro page provides a briefing regarding the data sheet of AIG. The HI page provides median and mean homozygosity intensity of AIGs associated with the studied ancestry groups. The results are shown according to the six analysis groups (AFR, AMR, EAS, EUR, SAS, and whole-continents) and followed by the population ancestry group. For each AIG, the median homozygosity intensity (mean homozygosity intensity shown in parenthesis) for each of population-ancestry groups are provided; the adjusted *P* value by using the false discovery rate is provided. The Pharmacogenomics (AIG) page provides the biological importance and drug information associated with AIGs of interest. Drug information is provided. In addition, on clicking the hyperlink Description of Columns, the contents, range of variables, and remarks of all variables are illustrated. On each page, significant *P* values are highlighted in red. Genetic Ancestry PhD provides catalogues of AIMs and AIGs associated with drugs and their related biological and pharmacogenetic information for global continents and populations.

Supplementary Note 3. — Top AIMs with the maximum difference in allele frequencies across the studied continental ancestry groups and within a specific continental ancestry group

SNVs rs10907223, rs7512595, and rs5065 were the top three AIMs with the maximum difference in allele frequencies across the five studied continental ancestry groups. The maximum differences were Δ = 0.437, 0.436, and 0.405 for each SNV, respectively. Rs10907223 was an AIM but not a PGx. The allele frequencies were 0.048, 0.079, 0.107, 0.116, and 0.485 in EUR, AMR, EAS, SAS, and AFR, respectively. Rs7512595 was identified as an AI-PGx responsible for the PK/PD of peginterferon alfa-2b and ribavirin for infectious liver disease caused by the hepatitis C Virus. The allele frequencies were 0.004, 0.029, 0.0764, 0.118, and 0.439 in EAS, SAS AMR, EUR, and AFR, respectively. Rs5065, which is stop lost SNV on *NPPA*, was an identified AI-PGx responsible for the PK/PD of amlodipine and chlorthalidone hypertension drugs. The allele frequencies were 0.013, 0.114, 0.123, 0.131, and 0.418 in EAS, AMR, EUR, SAS, and AFR, respectively. Drug targets and allele frequencies of the aforementioned AI-PGx are provided in our pharmacogenomic database.

SNVs rs2665582, rs4646440, and rs10210302 were the top three AIMs with the maximum difference in allele frequencies within a specific continent. SNV rs2665582 had the maximum difference in allele frequency (Δ = 0.422, adjusted $P = 7.948 \times 10^{-19}$ in Fisher's exact test), which was found within the American-ancestry group. Rs2665582 was an AIM but not a PGx. SNV rs4646440 had the second largest difference in allele frequency (Δ = 0.405, adjusted $P = 2.660 \times 10^{-19}$ in Fisher's exact test), which was found within the American-ancestry group. The allele frequencies were 0.077, 0.170, 0.219, and 0.482 in PUR CLM, MXL, and PEL, respectively. Rs4646440 was an AI-PGx responsible for the PK/PD of drug methadone. The details of the drug categories triggered by or associated with the AI-PGx can be accessed from our genetic ancestry pharmacogenomic database Genetic Ancestry PhD. Finally, SNV rs10210302 had the third largest difference in allele frequency (Δ = 0.397, adjusted $P = 1.331 \times 10^{-15}$ in Fisher's exact test) which was found within the American-ancestry group. The allele frequencies of *G* were 0.088, 0.258, 0.410, and 0.486 in PEL, MXL, CLM, and PUR respectively. Rs10210302 was an AI-PGx responsible for the PK/PD of the drug adalimumab.

Supplementary Figure 1. — Principal component analysis of whole-genome homozygosity intensity of 2,504 individuals.

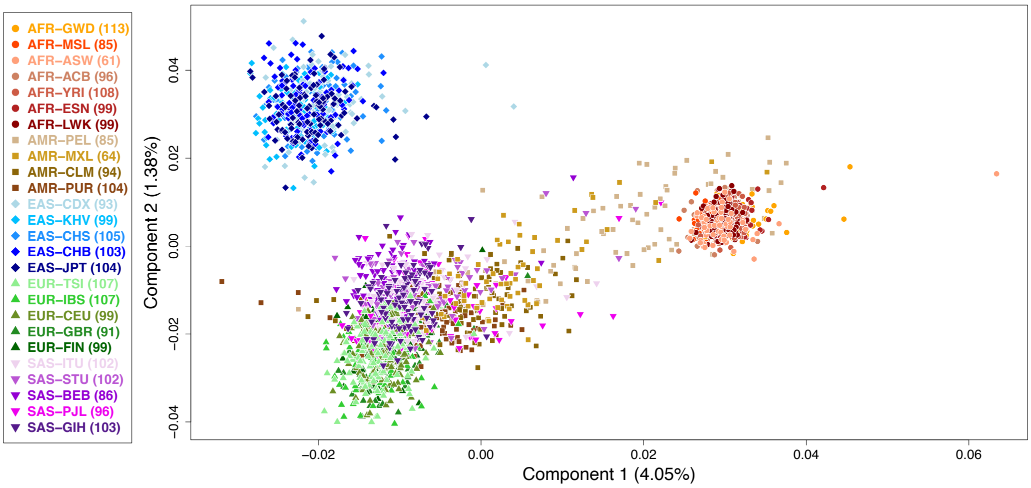

Supplementary Figure 2. — Parallel coordinates plot of 10-fold cross validations.

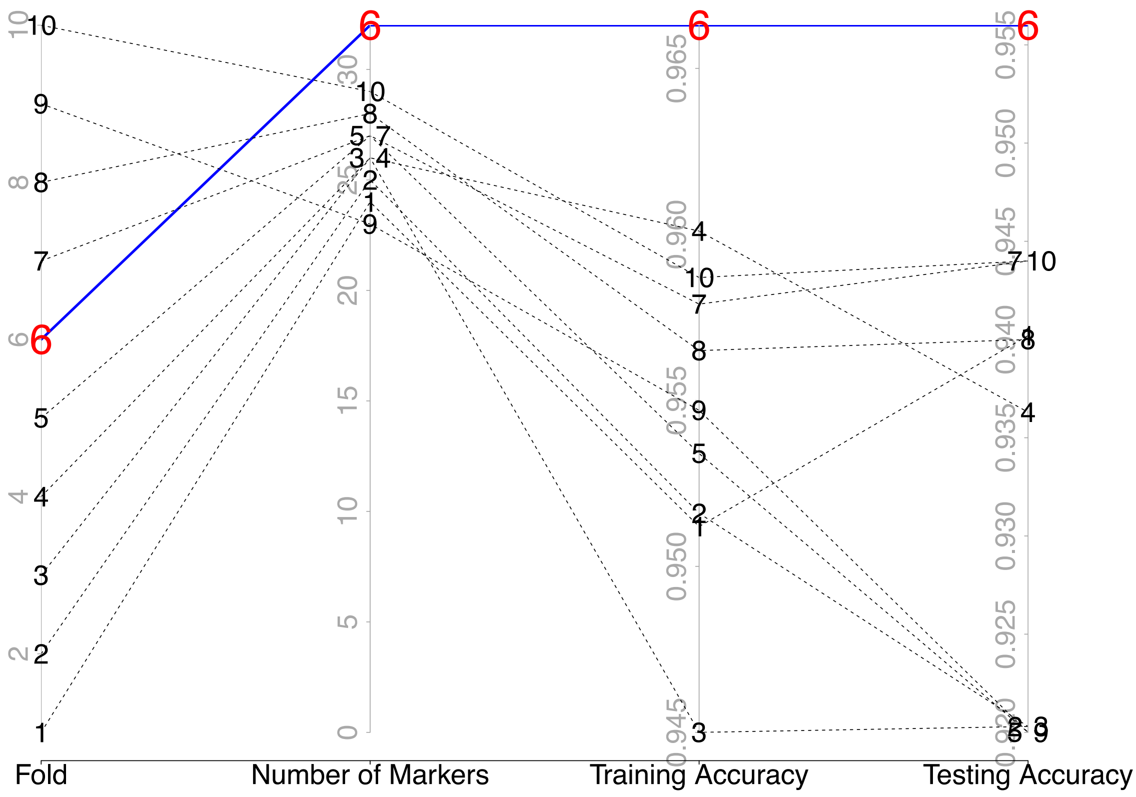

The sixth fold exhibits the highest training (0.965) and testing accuracy (0.955).

Supplementary Figure 3. — Overlay line graph of 10-fold cross validations for training and testing accuracy.

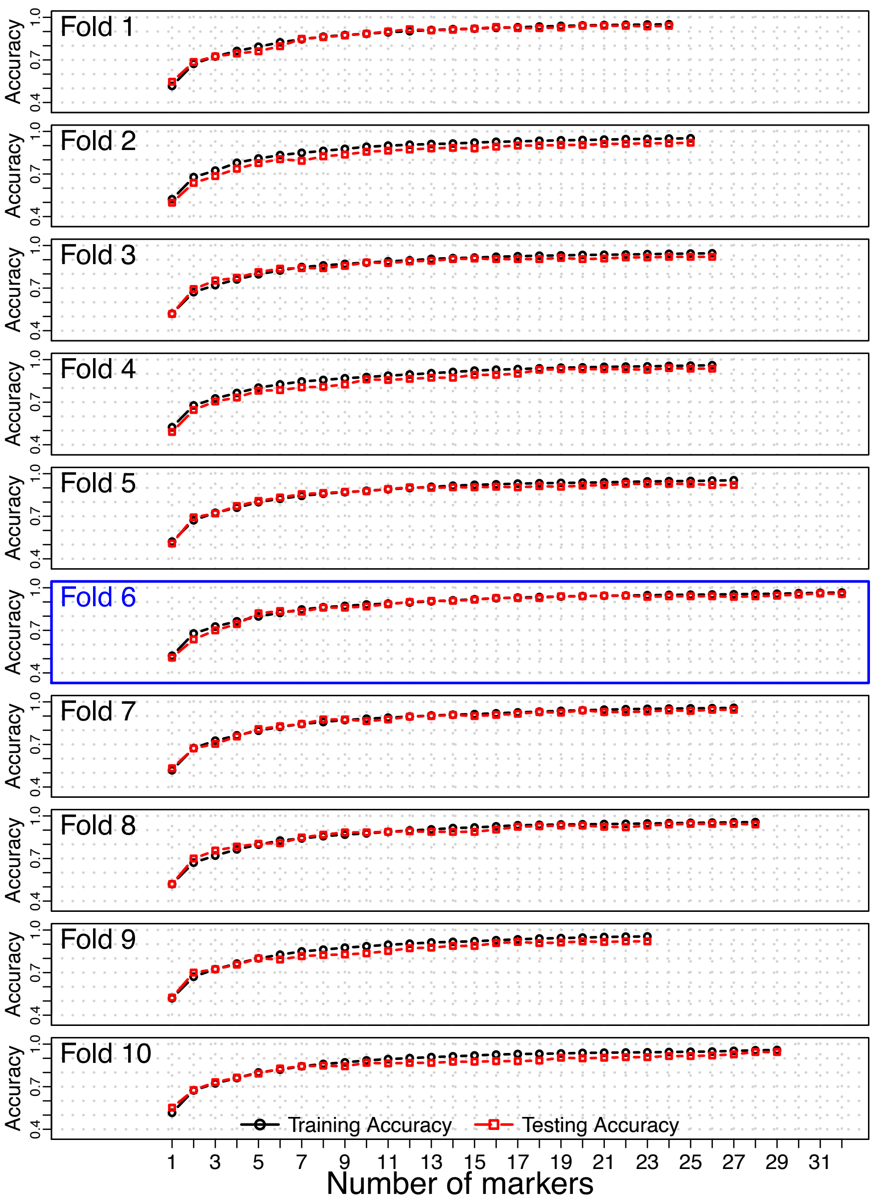

The sixth fold exhibits the highest training and testing accuracy and the best model contains 32 key predictors, including 31 ancestry-informative markers (AIMs) and 1 ancestry-informative gene (AIG).

Supplementary Figure 4. — Staked-bar/Box-whisker plot of the best prediction panel.

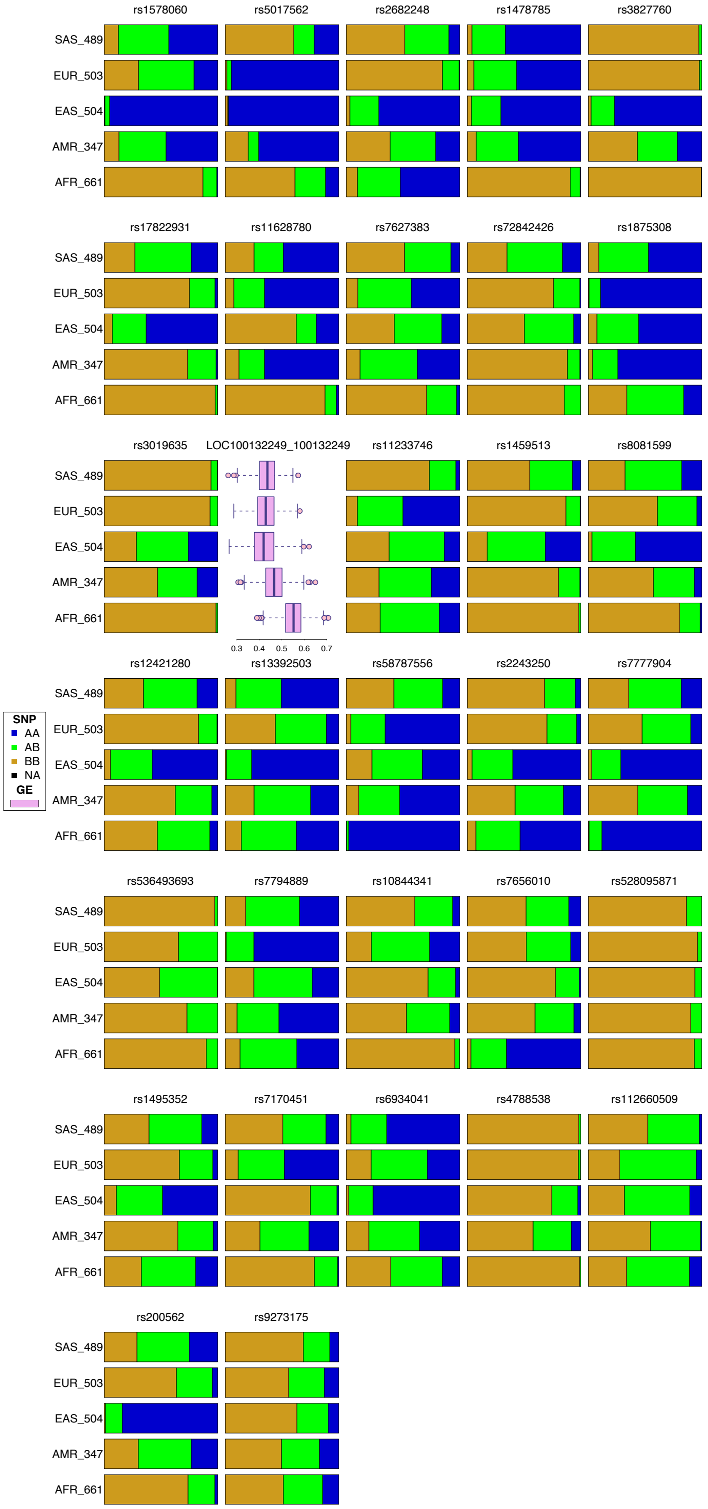

Distributions of genotypes from 31 ancestry-informative markers (AIMs) and homozygosity intensity from an ancestry-informative gene (AIG) are display

Supplementary Figure 5. — Marker impact plot of the best prediction panel.

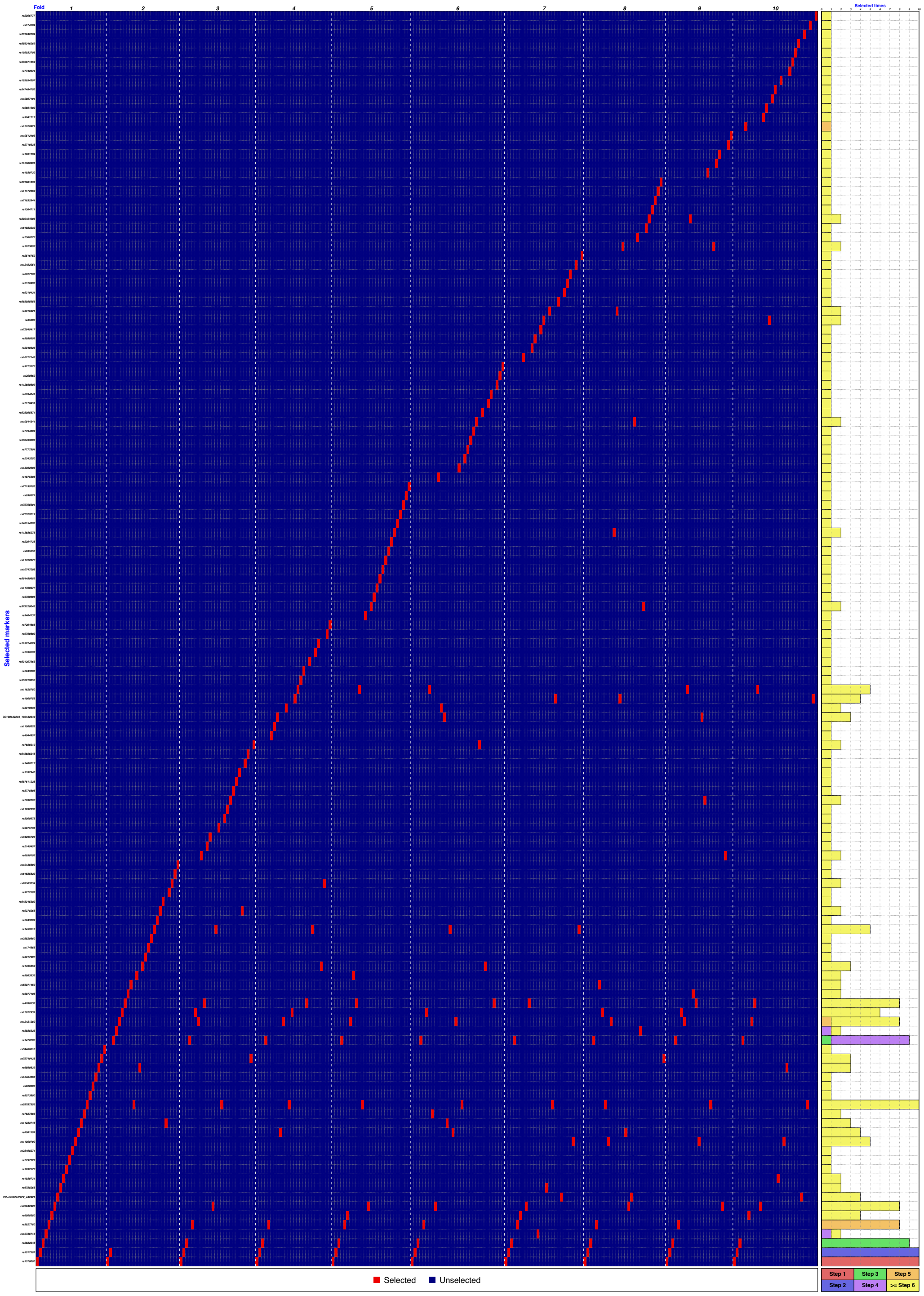

This plot summarizes the occurrence status and frequencies for each of the 31 key predictors in the 10-fold cross-validations. In the left panel, for each row (a predictor) and each column (a step of variable selection), each cell indicates an inclusion (red) or not (blue). In the right panel, a stacked bar displays the respective frequency that a specific predictor is included in a specific step of variable selection. Color is used to indicate the step in which a predictor is included in variable selection.

Supplementary Figure 6. — Multidimensional scaling plot of 2,504 individuals and 32 predictors.

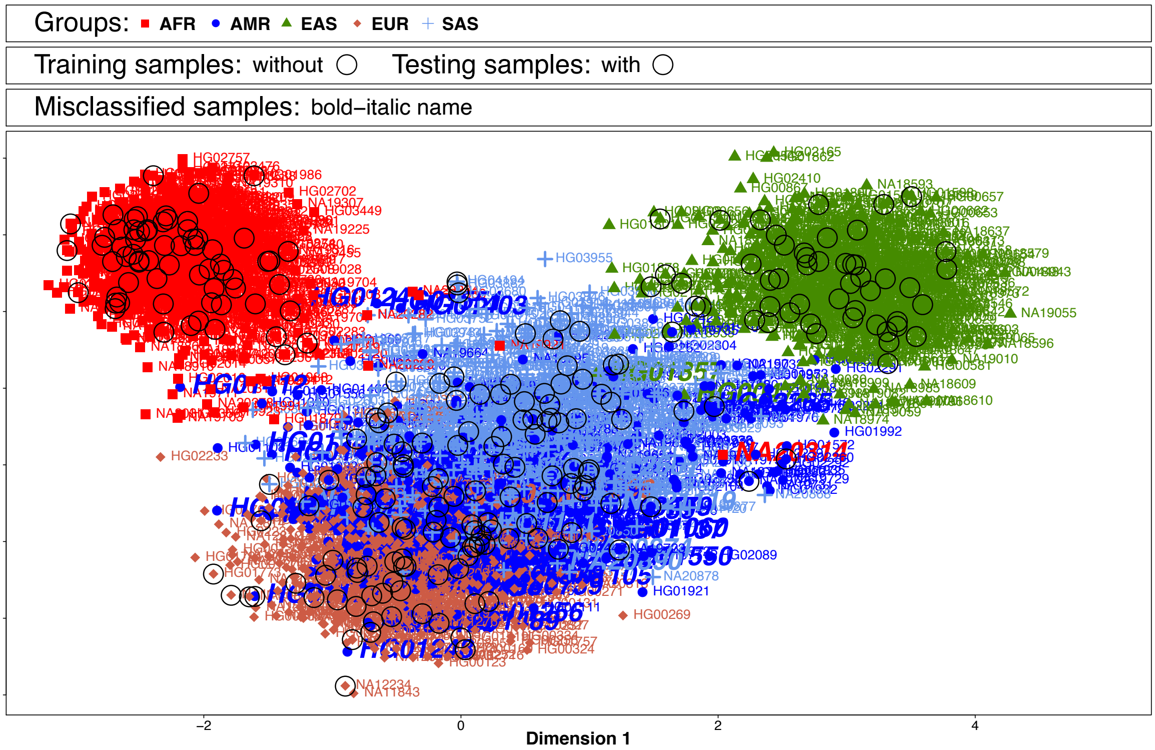

Supplementary Figure 7. — Ideograms of genome-wide homozygosity intensity.

a.

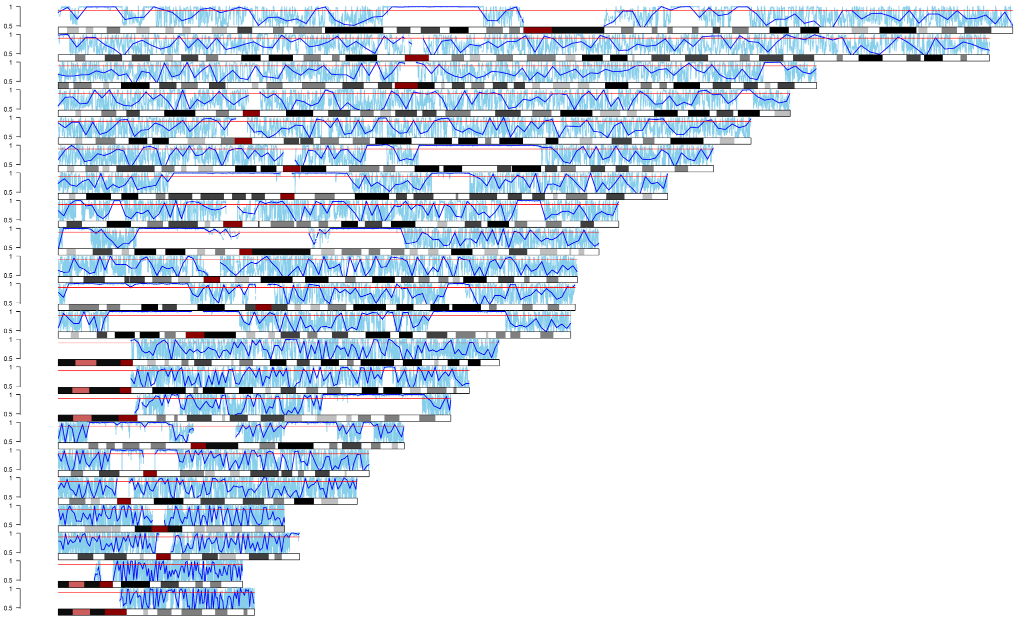

b.

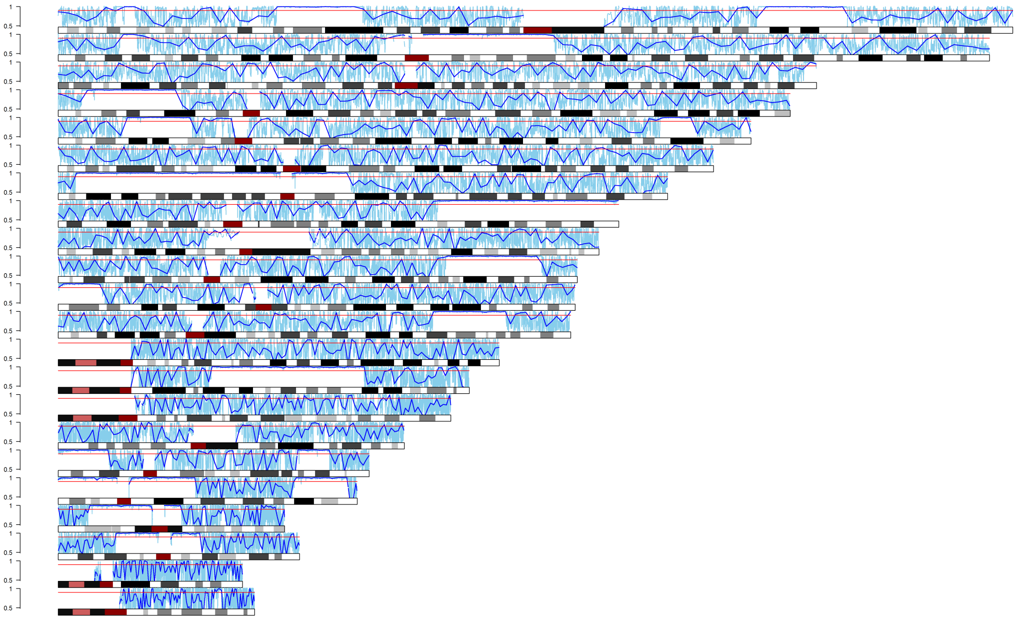

Each vertical axis represents homozygosity intensity from 0 to 1. The estimated curve (light blue) and smoothed curve (deep blue) of homozygosity intensity across the 22 autosomes are displayed. Chromosome bands near to the centromeric gaps are marked by red. **a.** HG04070 (ITU) who exhibited extremely long genomic segments under HD. **b.** HG02684 (CEU) who exhibited multiple genomic segments under homozygosity disequilibrium.

Supplementary Figure 8. — Global genotype distribution of rs1801133 on *MTHFR*.

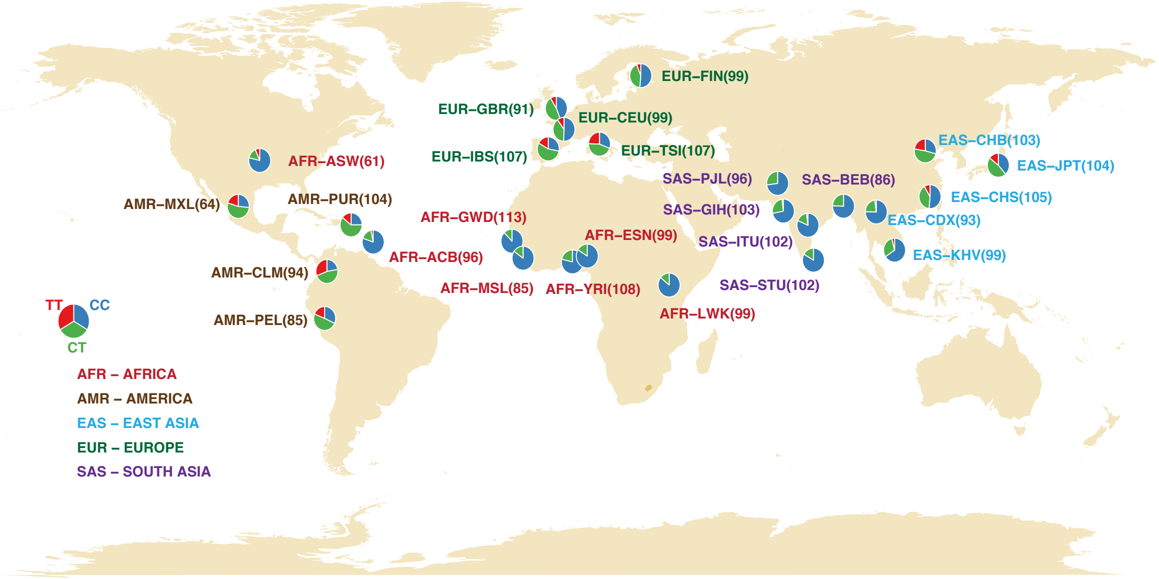

References:

1. Yang, H.C. *et al.* MPDA: Microarray pooled DNA analyzer. *BMC Bioinformatics* **9**, 196 (2008).
2. Yang, H.C. *et al.* A genome-wide study of preferential amplification/hybridization in microarray-based pooled DNA experiments. *Nucleic Acids Research* **34**, e106 (2006).
